# Supplementary material for: A family of Type VI secretion system effector proteins that form ion-selective pores
Source: Nat Commun. 2019 Dec 2;10:5484. doi: 10.1038/s41467-019-13439-0 (PMC6889166; doi:10.1038/s41467-019-13439-0)
Supplement: Supplementary file 2 — Description of Additional Supplementary Files [file 41467_2019_13439_MOESM2_ESM.pdf]

Description of Additional Supplementary Files

File Name: Supplementary Data 1

Description: Details of Ssp6 homologues identified using HMMER.
