# Supplementary material for: A family of Type VI secretion system effector proteins that form ion-selective pores
Source: Nat Commun. 2019 Dec 2;10:5484. doi: 10.1038/s41467-019-13439-0 (PMC6889166; doi:10.1038/s41467-019-13439-0)
Supplement: Supplementary file 4 — Reporting Summary [file 41467_2019_13439_MOESM4_ESM.pdf]

nature research

Corresponding author(s): Sarah Coulthurst

Last updated by author(s): Oct 31, 2019

Reporting Summary

Nature Research wishes to improve the reproducibility of the work that we publish. This form provides structure for consistency and transparency in reporting. For further information on Nature Research policies, see Authors & Referees and the Editorial Policy Checklist.

Statistics

For all statistical analyses, confirm that the following items are present in the figure legend, table legend, main text, or Methods section.

n/a	Confirmed	
☐	☒	The exact sample size (*n*) for each experimental group/condition, given as a discrete number and unit of measurement
☐	☒	A statement on whether measurements were taken from distinct samples or whether the same sample was measured repeatedly
☐	☒	The statistical test(s) used AND whether they are one- or two-sided<br>*Only common tests should be described solely by name; describe more complex techniques in the Methods section.*
☒	☐	A description of all covariates tested
☒	☐	A description of any assumptions or corrections, such as tests of normality and adjustment for multiple comparisons
☒	☐	A full description of the statistical parameters including central tendency (e.g. means) or other basic estimates (e.g. regression coefficient) AND variation (e.g. standard deviation) or associated estimates of uncertainty (e.g. confidence intervals)
☒	☐	For null hypothesis testing, the test statistic (e.g. *F*, *t*, *r*) with confidence intervals, effect sizes, degrees of freedom and *P* value noted<br>*Give P values as exact values whenever suitable.*
☒	☐	For Bayesian analysis, information on the choice of priors and Markov chain Monte Carlo settings
☒	☐	For hierarchical and complex designs, identification of the appropriate level for tests and full reporting of outcomes
☒	☐	Estimates of effect sizes (e.g. Cohen's *d*, Pearson's *r*), indicating how they were calculated

Our web collection on statistics for biologists contains articles on many of the points above.

Software and code

Policy information about availability of computer code

Data collection	Flow cytometry: FACS Diva software (BD Biosciences)<br>Electrophysiology: WinEDR V3.0.5
Data analysis	Microscopy: OMERO suite as described and referenced in Methods<br>Electrophysiology: WinEDR V3.8.6, TAC X V4.3.3, Clampex 10.2<br>Flow cytometry: FlowJo V 10.4.2<br>Identification and analysis of Ssp6 homologues: Prokka v1.13.3, HMMER suite (v 3.1b2), MUSCLE (v3.8.31), IQTREE (v1.6.5), ggtree (v1.15.6), ggplot2 (v3.1.1), gggenes (v0.3.2) all referenced in Methods; wrapper scripts available at GitHub (https://github.com/djw533/ssp6-paper/tree/master/scripts).<br>GraphPad Prism 7, GraphPad Prism 8

For manuscripts utilizing custom algorithms or software that are central to the research but not yet described in published literature, software must be made available to editors/reviewers. We strongly encourage code deposition in a community repository (e.g. GitHub). See the Nature Research guidelines for submitting code & software for further information.

Data

Policy information about availability of data

All manuscripts must include a data availability statement. This statement should provide the following information, where applicable:

- Accession codes, unique identifiers, or web links for publicly available datasets
- A list of figures that have associated raw data
- A description of any restrictions on data availability

All data supporting the findings of this study are available within the paper and its supplementary information files.

Field-specific reporting

Please select the one below that is the best fit for your research. If you are not sure, read the appropriate sections before making your selection.

☒ Life sciences ☐ Behavioural & social sciences ☐ Ecological, evolutionary & environmental sciences

For a reference copy of the document with all sections, see nature.com/documents/nr-reporting-summary-flat.pdf

Life sciences study design

All studies must disclose on these points even when the disclosure is negative.

Sample size	No sample size calculations were performed
Data exclusions	No exclusions
Replication	Attempts at replication were successful
Randomization	n/a
Blinding	n/a

Reporting for specific materials, systems and methods

We require information from authors about some types of materials, experimental systems and methods used in many studies. Here, indicate whether each material, system or method listed is relevant to your study. If you are not sure if a list item applies to your research, read the appropriate section before selecting a response.

Materials & experimental systems

n/a	Involved in the study	
☐	☒	Antibodies
☒	☐	Eukaryotic cell lines
☒	☐	Palaeontology
☒	☐	Animals and other organisms
☒	☐	Human research participants
☒	☐	Clinical data

Methods

n/a	Involved in the study	
☒	☐	ChIP-seq
☐	☒	Flow cytometry
☒	☐	MRI-based neuroimaging

Antibodies

Antibodies used

Polyclonal anti-Hcp serum (Rabbit, Eurogentec)
Polyclonal anti-TssL serum (Rabbit, Eurogentec)
Polyclonal anti-OmpA serum (Rabbit, gift from Roland Freudl lab)
Monoclonal anti-HA (Mouse, clone 12CA5, raised against YPYDVPDYA. Generon or MRC-PPU Reagents, University of Dundee)
Monoclonal anti-FLAG (Mouse, clone M2, raised against DYKDDDDK, Sigma).
Monoclonal anti-EFTu (Mouse, clone 900, Hycult Biotech)
Monoclonal anti-MBP (Mouse, cell line B48, New England Biolabs)
Secondary antibodies were peroxidase-conjugated goat anti-mouse (cat# 170-6516) and goat anti-rabbit secondary (# 170-6515) antibodies (Bio-Rad).

Validation

Anti-Hcp is a custom polyclonal antiserum that we have previously validated in Serratia marcescens (Murdoch et al., 2011)
Anti-TssL is a custom polyclonal antiserum that has been validated within this study (Supplementary Figure 10)
Polyclonal anti-OmpA was previously validated in Escherichia coli (Donald et al., 2008)
Monoclonal anti-HA is a commercial antibody has been previously validated in Serratia marcescens (Ostrowski et al., 2018)
Monoclonal anti-FLAG is a commercial antibody has been previously validated in Serratia marcescens (Cianfanelli et al., 2016)
Monoclonal anti-EFTu is a commercial antibody previously used in various Gram-negative bacteria including Serratia marcescens (Ostrowski et al., 2018) and Pseudomonas aeruginosa (Kunert et al., 2007)
Monoclonal anti-MBP is a commercial antibody previously validated in Serratia marcescens (Diniz et al., 2015)

Flow Cytometry

Plots

Confirm that:

☒ The axis labels state the marker and fluorochrome used (e.g. CD4-FITC).

☒ The axis scales are clearly visible. Include numbers along axes only for bottom left plot of group (a 'group' is an analysis of identical markers).

☒ All plots are contour plots with outliers or pseudocolor plots.

☒ A numerical value for number of cells or percentage (with statistics) is provided.

Methodology

Sample preparation	Sample preparation for flow cytometry is described in Methods
Instrument	LRS Fortessa (BD Biosciences)
Software	Data were collected using the software FACSDiva (BD Biosciences).<br>Analysis of data was performed using the software FlowJo v10.4.2 (Treestar Inc.)
Cell population abundance	For all experiments 50.000 events were collected
Gating strategy	Gating strategy is described in the legend for Supplementary Figure 3.

☒ Tick this box to confirm that a figure exemplifying the gating strategy is provided in the Supplementary Information.
